# Hemostatic Changes Following Red Blood Cell Transfusion in Critically Ill Patients: A Retrospective Cohort Study

**DOI:** 10.3390/jcm14228048

**Published:** 2025-11-13

**Authors:** Piotr F. Czempik

**Affiliations:** 1 Department of Anesthesiology and Intensive Care, Faculty of Medical Sciences in Katowice, Medical University of Silesia, 40-752 Katowice, Poland; pczempik@sum.edu.pl; 2 Transfusion Committee, University Clinical Center of Medical University of Silesia in Katowice, 40-752 Katowice, Poland

**Keywords:** anemia, hemostasis, intensive care unit, red blood cells, rotational thromboelastometry, transfusion

## Abstract

**Background/Objectives**: Red blood cells actively influence hemostasis by enhancing platelet activation, promoting thrombin generation, and contributing to clot structure. Their transfusion may alter coagulation dynamics, yet conventional tests often miss these effects, highlighting the need for viscoelastic monitoring. **Methods**: This retrospective single-center study carried out in the intensive care unit analyzed ROTEM, conventional coagulation tests, and CBC data pre–post-single-unit RBC transfusion. Platelet and fibrinogen contributions to clot strength were assessed. Statistical comparisons used the Wilcoxon signed-rank test, with significance set at *p* < 0.05. Ethical approval was waived. **Results**: Thirty-five patients were analyzed; ROTEM revealed reduced fibrinogen contribution to clot strength and decreased hyperfibrinolysis post-transfusion. Conventional tests showed minimal changes, except for a significant increase in D-dimer levels. **Conclusions**: Transfusion of a single RBC in non-bleeding critically ill patients with severe anemia may lead to diminished fibrinogen-based clot architecture or fibrin cross-linking, as well as a decrease in hyperfibrinolysis. Most of the hemostatic effects of RBC transfusion cannot be detected by conventional coagulation tests. The net effect of RBC transfusion remains undetermined and requires further mechanistic studies.

## 1. Introduction

Red blood cells (RBCs) have long been considered passive elements in the circulatory system, primarily responsible for oxygen transport. However, recent advances in hematology and vascular biology have revealed that RBCs play dynamic and multifaceted roles in hemostasis. Far from being inert bystanders, RBCs actively contribute to the regulation of clot formation, thrombin generation, and thrombus architecture, influencing both physiological and pathological coagulation processes.

One of the most striking mechanical contributions of RBCs is their role in platelet margination. In flowing blood, RBCs tend to occupy the central axial stream, displacing platelets toward the periphery of the vessel lumen. This redistribution enhances platelet contact with the endothelial surface, particularly at sites of vascular injury, thereby facilitating platelet adhesion and activation [1]. Although this phenomenon is well-documented in vivo, it remains difficult to replicate under static in vitro conditions due to the absence of shear forces and flow dynamics.

In addition to mechanical effects, RBCs engage in biochemical crosstalk with platelets. They release adenosine 5′-diphosphate (ADP), a potent agonist that triggers platelet activation and aggregation. RBCs also produce thromboxane A2 (TXA2), further amplifying platelet responsiveness. These interactions are particularly relevant in the context of stored or transfused RBCs, which may exhibit altered membrane properties and increased expression of adhesion molecules such as CD47 and ICAM-4. These molecules can bind to platelet receptors, promoting heterotypic cell–cell adhesion and potentially contributing to a prothrombotic state [1].

Moreover, RBCs influence platelet activity indirectly through nitric oxide (NO) scavenging. Hemoglobin within RBCs binds to NO, a vasodilator and inhibitor of platelet activation. By reducing NO bioavailability, RBCs create a microenvironment that favors platelet aggregation and vasoconstriction, further tipping the balance toward thrombosis [2].

Beyond platelet interactions, RBCs also modulate coagulation cascade dynamics. A key mechanism involves the externalization of phosphatidylserine (PS) on the RBC membrane, particularly under stress conditions such as storage, oxidative damage, or apoptosis [3]. Phosphatidylserine exposure provides a negatively charged surface that facilitates the assembly of the prothrombinase complex, accelerating the conversion of prothrombin to thrombin—a central event in clot formation. This procoagulant activity is further amplified by RBC-derived microparticles (MPs), which are submicron vesicles shed from the RBC membrane [4]. Microparticles not only carry PS but may also express tissue factor (TF), enabling activation of both the intrinsic and extrinsic coagulation pathways [5,6]. Notably, MPs are released in greater quantities from allogeneic or stored RBCs, likely due to membrane rigidity and reduced deformability, which are hallmarks of storage lesions [7].

The cumulative effect of these interactions is reflected in thrombin generation assays, which integrate the contributions of plasma coagulation factors, platelets, and cellular elements such as RBCs and MPs. Thrombin generation serves as a global marker of hemostatic potential and is increasingly used to assess hypercoagulability in clinical settings.

Finally, RBCs are physically incorporated into developing thrombi, where they influence the density, elasticity, and mechanical stability of the clot [8]. Their presence affects fibrin architecture and can alter the susceptibility of thrombi to fibrinolysis. RBC-rich clots tend to be more resistant to breakdown, which may have implications for thrombotic risk and therapeutic strategies.

Given the growing recognition of RBCs as active modulators of hemostasis, it is critical to understand how RBC transfusion—a common intervention in critical care—affects coagulation dynamics. While transfusion is often guided by hemoglobin thresholds, its impact on thrombin generation and clot stability remains underexplored, particularly in non-bleeding critically ill patients, where the balance between thrombosis and hemostasis is delicate.

This study aims to investigate the hemostatic consequences of single-unit RBC transfusion in this vulnerable population, using advanced coagulation assays to capture subtle shifts in thrombin generation and platelet function.

## 2. Materials and Methods

### 2.1. Study Design and Setting

This was a retrospective, observational, single-center study conducted in the mixed medical–surgical intensive care unit (ICU) of a large academic tertiary care hospital affiliated with the Medical University of Silesia in Katowice, Poland. A heterogeneous population of critically ill adult patients is cared for in the local ICU, including those with medical, surgical, trauma, and perioperative conditions. The ICU is equipped with advanced hemodynamic monitoring, bedside laboratory diagnostics, and point-of-care coagulation testing, including viscoelastic hemostatic assays (VHAs), namely rotational thromboelastometry (ROTEM).

### 2.2. Data Source and Extraction

Patient data were extracted from the hospital’s integrated electronic medical record system (Asseco Medical Management System, AMMS; Asseco Medical Solutions, Rzeszów, Poland). The electronic medical records include comprehensive clinical, laboratory, and transfusion records, allowing for accurate temporal correlation between interventions and laboratory measurements. Data extraction was performed by trained research personnel using standardized data collection forms to ensure consistency and minimize transcription errors.

### 2.3. Variables Collected

The following categories of data were collected:

- Demographic data: age, sex, body weight, total blood volume—calculated as weight [kg] × 65 mL (women)/75 mL (men);
- Clinical data: ICU admission diagnosis; date and time of RBC transfusion, indication for transfusion (e.g., anemia without active bleeding), type of RBC (leukoreduced, irradiated, etc.), RBC volume transfused, RBC volume as percent of the patient’s total blood volume, and RBC age (storage duration) at the moment of transfusion;
- Laboratory data:
  1. Rotational thromboelastometry (ROTEM Delta, TEM Innovations GmbH, Munich, Germany). The assays routinely performed in the local ICU included INTEM (assessment of the intrinsic coagulation pathway), EXTEM (assessment of the extrinsic coagulation pathway), FIBTEM (assessment of fibrinogen-based clot firmness through inhibition of platelets)*,* and APTEM (detection of hyperfibrinolysis and assessment of the effect of antifibrinolytic agents). The analyzed parameters included the following: clotting time (CT), clot formation time (CFT), alpha angle (AA), maximum clot firmness (MCF), maximal lysis (ML), lysis index at 30 min (LI30), and lysis index at 45 min (LI45). Platelet contribution to clot strength was estimated by calculating the difference in MCF between the EXTEM and FIBTEM assays.
  2. Conventional coagulation tests (CCTs) included the following: prothrombin time (PT), international normalized ratio (INR), prothrombin activity (%), activated partial thromboplastin time (aPTT), thrombin time (TT), D-dimer (DD), and fibrinogen concentration measured with the Clauss method.
  3. Complete Blood Count (CBC): hemoglobin (Hb) concentration and platelet count (PLT).

All conventional laboratory tests were performed in the hospital’s central laboratory using standardized protocols and calibrated equipment. Viscoelastic assays were performed at the bedside by trained ICU staff.

### 2.4. Inclusion and Exclusion Criteria

Patients were eligible for inclusion if they met the following criteria:

- Age ≥ 18 years;
- Hospitalized in the ICU;
- Received a single unit of RBC transfusion for the indication of anemia (defined as hemoglobin < 8 g dL^−1^) without active bleeding per attending physician’s discretion;
- Availability of ROTEM results within 1 h pre- and post-RBC transfusion.

Exclusion criteria included the following:

- Receipt of other blood components (e.g., plasma, platelets, cryoprecipitate, fibrinogen concentrate) during the same time window;
- Known coagulation disorders or use of anticoagulants that could confound ROTEM results.

### 2.5. Endpoints and Outcome Measures

The primary endpoint was the change in ROTEM parameters post-single-unit RBC transfusion. Secondary endpoints included changes in CCTs and PLT.

### 2.6. Statistical Analysis

Statistical analysis was performed using licensed statistical software (Stata 18 Basic Edition; StataCorp LLC, College Station, TX, USA). Continuous variables were assessed for normality using the Shapiro–Wilk test. Continuous variables with non-normal distribution were presented as median (Me) and interquartile range (IQR). Categorical variables were presented as numbers (n) and percentages (%). Comparisons of paired laboratory parameters pre–post-RBC transfusion were conducted using the non-parametric Wilcoxon signed-rank test. Comparisons of continuous variables between subgroups were performed using the Wilcoxon rank-sum test. To account for the possible confounders multivariate linear regression analysis was performed and coefficients and their 95% confidence intervals (CIs) calculated. A two-tailed *p*-value < 0.05 was considered statistically significant. No imputation was performed for missing data, and only complete cases (ROTEM parameters) were included in the final analysis.

### 2.7. Ethical Considerations

Given the retrospective nature of this study and the use of anonymized data extracted from routine clinical records, the Bioethics Committee of the Medical University of Silesia in Katowice (Poland) reviewed the study protocol and waived the requirement for formal ethical approval and informed consent. The study was conducted in accordance with the Declaration of Helsinki and institutional guidelines for research involving human subjects.

## 3. Results

There were 35 patients with complete pre–post-RBC transfusion ROTEM results—from October 2023 to November 2025. We excluded one patient who was missing FIBTEM assay results post-transfusion. Blood for CCTs and ROTEM was drawn directly before the start of RBC transfusion and after its completion.

### 3.1. Study Population Characteristics

The characteristics of the study population are presented in Table 1.

Almost all RBCs were leucoreduced (n = 34, 97.1%). The median RBC age at the moment of transfusion was 18.0 (15.0–26.0) days. The median RBC volume was 250.0 (250.0–300.0) mL, which constituted 4.3 (IQR 3.7–5.5) % of patients’ total blood volume.

### 3.2. Results of Conventional Coagulation Tests Pre–Post-Red Blood Cell Transfusion

The results of CCTs pre–post-RBC transfusion are presented in Table 2.

The only statistically significant difference pre–post-transfusion was an increase in DD concentration, which was highly elevated both pre- and post-transfusion. Prothrombin time was above the reference range both pre- and post-transfusion, whereas prothrombin activity was below the reference range. Notably, fibrinogen concentration was highly elevated both pre- and post-transfusion.

### 3.3. Results of Rotational Thromboelastometry Pre–Post-Red Blood Cell Transfusion

Viscoelastic assay results pre–post-RBC transfusion are presented in Table 3.

Several statistically significant differences in ROTEM parameters pre–post-RBC transfusion were observed. Increases of CFT in EXTEM and FIBTEM were observed. A decrease in INTEM MCF was observed, although this parameter exceeded the upper reference range both pre- and post-transfusion, which may reflect high fibrinogen concentration in the study group. A significant decrease in EXTEM ML was observed, which was the most significant percent change among the analyzed parameters—it dropped by 35.0%. Significant increases were observed in both EXTEM LI30 and EXTEM LI45—by 9.7 and 13.8%, respectively. A significant decrease in FIBTEM MCF post-transfusion was observed, although again the value was above the reference range both pre- and post-transfusion. The percent change in FIBTEM MCF was less significant than for EXTEM ML—it dropped by 5.7%. A decrease in APTEM MCF was observed—by 2.6%. As to the contribution of PLT to clot strength, although a significant increase in MCF EXTEM-FIBTEM post-transfusion was observed—by 8.1%—this was primarily due to a decrease in FIBTEM MCF post-transfusion.

In order to account for possible confounders, an analysis of changes in ROTEM parameters post-transfusion in the subgroups of patients who received fresher and older RBCs was performed (Table 4).

Of all ROTEM parameters the only change pre–post-transfusion that was found to be significantly different between patients who received fresher and older RBCs was FIBTEM CT.

The other possible confounders for the observed changes in ROTEM parameters post-transfusion (Table 3) could be the dilution effect of RBC volume and admission diagnosis; therefore, multivariate regression analysis was performed. The multivariate linear regression analysis of predictors of change in FIBTEM MCF is presented in Table 5, whereas that for change in MCF EXTEM-FIBTEM is shown in Table 6.

It has been shown that RBC age and its volume effect, as well as admission diagnosis, were not predictors of change in FIBTEM MCF and MCF EXTEM-FIBTEM. As far as MCF EXTEM-FIBTEM is concerned, its change was due to changes in FIBTEM MCF, not the number of PLTs.

## 4. Discussion

This study provides compelling evidence that even a single-unit RBC transfusion can induce measurable changes in hemostatic function in non-bleeding critically ill patients, which was detected by VHA, specifically ROTEM. Changes in INTEM MCF, EXTEM CFT, FIBTEM CFT, FIBTEM MCF, and APTEM MCF suggest decreases in the contribution of fibrinogen to clot propagation and clot firmness post-RBC transfusion in the study group. A significant increase in MCF difference between EXTEM and FIBTEM post-transfusion was observed, which was primarily due to a decrease in FIBTEM MCF post-RBC transfusion; linear regression analysis showed that PLT number was not a predictor of this parameter change. The most pronounced change—the drop in EXTEM ML—suggests that hyperfibrinolysis decreased post-RBC transfusion. Therefore, observed alterations include diminished fibrinogen-based clot architecture or fibrin cross-linking and reduced hyperfibrinolysis post-RBC transfusion. These findings challenge the traditional view of RBC transfusion as a purely oxygen-delivery intervention and underscore its broader impact on coagulation dynamics.

In terms of the quantitative impact of RBC transfusion on fibrinogen function, a decrease of 2.0 mm in FIBTEM MCF post-transfusion equates to a deficit of approximately 50.0 mg/kg of fibrinogen according to ROTEM-based fibrinogen concentrate dosing algorithms [9]. This observation highlights the superior sensitivity of VHAs in detecting functional deficits that may be missed by antigen-based assays like the Clauss method, which showed no significant change in fibrinogen concentration post-transfusion. The discrepancy between functional and antigenic measurements reinforces the need for dynamic, real-time monitoring tools in transfusion management.

From a mechanistic standpoint, the reduction in fibrinogen function was not attributed to the dilution effect because one of the markers of dilution, the concentration of fibrinogen, the most abundant coagulation factor, did not change pre–post-RBC transfusion. The fact that fibrinogen concentration did not change pre–post-transfusion also indicates that continuous infusions of medications had no dilution effect on the concentration of coagulation factors. The indication for RBC transfusion was anemia, not bleeding; therefore, the patients in this study were not receiving fluid resuscitation. Impact of dilution as a confounder was also dismissed by performing linear regression analysis. Possible reasons for reduction in fibrinogen function post-transfusion could be RBC-induced alterations in plasma protein interactions, or changes in fibrin polymerization kinetics. Stored RBCs are known to release MPs and bioactive lipids that can interfere with fibrin assembly, potentially impairing clot integrity. Additionally, RBCs may modulate thrombin generation, which in turn affects fibrinogen cleavage and polymerization. These interactions are complex and multifactorial, warranting further investigation in both in vitro and in vivo models.

Clinically, this finding has direct implications for transfusion protocols during hemorrhage. It suggests that RBC transfusion should not be viewed in isolation but rather as part of a broader hemostatic strategy. Monitoring fibrinogen function post-transfusion is essential, especially in patients with borderline fibrinogen levels or ongoing bleeding. In our institution, ROTEM is frequently performed pre–post-transfusion of blood components, including RBCs, to guide targeted therapy and prevent under-treatment of coagulopathy.

The observed increase in DD concentration post-transfusion suggests more pronounced fibrinolytic activity. While DD is not a specific marker of fibrinolysis [10], it reflects the breakdown of cross-linked fibrin and serves as a surrogate for plasmin activity. The increase in DD may indicate increased fibrin turnover. On the contrary, ROTEM showed decreased hyperfibrinolysis post-transfusion. The reason for increasing DD concentration and decreasing hyperfibrinolysis post-transfusion may be the fact that older RBCs contain MP, free Hb, and oxidative stress mediators that can activate coagulation pathways. This may lead to localized clot formation and degradation, increasing DD without triggering systemic hyperfibrinolysis. Decreased hyperfibrinolysis post-transfusion potentially may be mediated by RBC incorporation into thrombi. The other mechanism behind decreased hyperfibrinolysis is modulation of tissue plasminogen activator (t-PA) bound to RBCs and type 1 plasminogen activator inhibitor (PAI-1) in endothelial cells. Red blood cell-bound t-PA shows reduced systemic fibrinolytic activity but enhanced clot-targeted action, potentially minimizing hyperfibrinolysis. Byproducts of RBC breakdown (MPs, Hb, heme) may upregulate PAI-1 expression in endothelial cells, tipping the balance toward antifibrinolysis [11]. It raises the possibility that RBC transfusion may exert a stabilizing influence on the fibrin matrix, either through mechanical reinforcement or biochemical signaling. However, the exact mechanisms remain speculative and merit further exploration. This effect is particularly intriguing given the absence of antifibrinolytic therapy in the studied population. Future studies should consider measuring additional fibrinolytic markers, such as plasmin–antiplasmin complexes or t-PA levels, to elucidate the pathways involved.

The impact of RBC on hemostasis, whether fresher or older RBCs were transfused, was not shown to be different. The subgroup analysis revealed that only the change in one ROTEM parameter post-transfusion was different between subgroups, namely FIBTEM CT. This difference is very difficult to interpret because FIBTEM CT is impacted by several clotting factors (X, V, II, fibrinogen). The fact that no changes in FIBTEM parameters assessing clot propagation and firmness post-transfusion were different between subgroups may indicate that fibrinogen function is not the reason for this differential impact of fresher and older RBCs on FIBTEM CT. Moreover, I performed linear regression analysis to check if RBC age is a predictor of FIBTEM CT change post-transfusion, which showed a lack of statistical significance (*p* = 0.37).

This study adds to the growing recognition that RBC transfusion is not a neutral intervention but one that can modulate multiple aspects of hemostasis. In the context of critical illness, where patients often have complex coagulopathies and are at risk for both bleeding and thrombosis, understanding these effects is essential. The findings support a more nuanced approach to transfusion, one that incorporates functional assays, individualized thresholds, and real-time monitoring. Clinicians should consider the impact of RBC transfusion on patients with borderline fibrinogen concentration or function, and incorporate this effect into transfusion decision-making and hemostatic monitoring. Moreover, this study highlights the need for interdisciplinary collaboration between intensivists, hematologists, and transfusion specialists to optimize patient care. As VHAs become more widely available, their integration into transfusion protocols can enhance decision-making, reduce unnecessary component use, and improve outcomes.

Prospective, multicenter studies with larger cohorts are needed to validate these findings and explore their applicability across different patient populations. Investigations into the molecular mechanisms underlying RBC-induced changes in coagulation could yield novel therapeutic targets or biomarkers. Additionally, studies comparing the effects of leukoreduced versus non-leukoreduced units and different transfusion volumes would provide valuable insights into optimizing transfusion practices.

Several limitations of this study must be acknowledged. The retrospective design limits causal inference and control over confounding variables. Although efforts were made to isolate the effect of RBC transfusion by timing laboratory assessments pre–post-transfusion, unmeasured factors such as fluid shifts, medication effects, or underlying disease progression may have influenced the results. The single-center nature of the study further restricts generalizability, as the patient population consisted exclusively of critically ill individuals in an intensive care setting. Selection bias is also a concern, as only patients with complete ROTEM data were included. This may have skewed the sample toward those with more intensive monitoring or more severe illness; however, the decision to perform ROTEM analysis pre–post-RBC transfusion is left in our department to the attending clinician. The relatively small sample size limits statistical power and increases the risk of type II error, potentially obscuring subtle but clinically relevant changes; the absence of statistically significant findings for certain parameters should not be interpreted as definitive evidence of no effect, but rather as inconclusive due to limited power. Viscoelastic hemostasis assays themselves have inherent limitations. As in vitro assays, they cannot replicate the full complexity of in vivo hemostasis, particularly the contributions of endothelial cells, flow dynamics, and cellular interactions. The calculated platelet contribution (MCF EXTEM − FIBTEM) is an indirect measure and may not fully reflect true platelet function.

## 5. Conclusions

Transfusion of a single RBC in non-bleeding critically ill patients with severe anemia may lead to diminished fibrinogen-based clot architecture or fibrin cross-linking, as well as a decrease in hyperfibrinolysis. Most of the hemostatic effects of RBC transfusion cannot be detected by conventional coagulation tests. The net effect of RBC transfusion remains undetermined and requires further mechanistic studies.

## Figures and Tables

**Table 1:** Study population characteristics.

Characteristic	Value
Male sex [n (%)]	20 (57.1)
Age, Me ^1^ (IQR ^2^) [years]	62.0 (47.0–71.0)
Weight, Me (IQR) [kilograms]	87.0 (74.0–106.0)
Total blood volume, Me (IQR) [mL]	6300 (5200–7125)
Diagnosis:	
Post-surgery, n (%)	20 (57.1)
Acute respiratory failure, n (%)	5 (14.3)
Severe acute pancreatitis, n (%)	5 (14.3)
Subarachnoid hemorrhage, n (%)	2 (5.7)
Sepsis, n (%)	2 (5.7)
Sudden cardiac arrest, n (%)	1 (2.9)
C-reactive protein, Me (IQR) [mg L^−1^]	143.0 (93.1–237.0)
Creatinine, Me (IQR) [mg dL^−1^]	1.3 (0.7–2.2)
Blood urea nitrogen, Me (IQR) [mg dL^−1^]	37.8 (29.0–84.6)
Urea, Me (IQR) [mg dL^−1^]	80.9 (62.1–181.0)
Bilirubin, Me (IQR) [mg dL^−1^]	0.7 (0.3–1.3)
Hb ^3^ pre-transfusion [g L^−1^]	63.0 (61.0–70.0)
Hb post-transfusion [g L^−1^]	78.0 (72.0–81.0)

^1^ Median value. ^2^ Interquartile range. ^3^ Hemoglobin concentration.

**Table 2:** Results of conventional coagulation tests pre–post-red blood cell transfusion in the study group.

Parameter	Value, Me ^1^ (IQR ^2^) Pre-Transfusion	Value, Me (IQR) Post-Transfusion	*p*-Value	Reference Range
Prothrombin time [s]	13.8 (12.7–14.9)	13.9 (12.6–14.8)	0.53	9.4–12.5
International normalized ratio	1.14 (1.05–1.24)	1.15 (1.04–1.23)	0.68	0.80–1.20
Prothrombin activity [%]	76.0 (71.0–88.0)	76.0 (71.0–88.0)	1.00	80.0–120.0
Activated partial thromboplastin time [s]	35.2 (31.3–37.9)	33.9 (30.5–39.1)	0.69	25.4–36.9
Thrombin time [s]	17.1 (15.9–19.2)	16.6 (15.9–18.1)	0.28	10.3–16.6
**D-dimers [ng mL^−1^]**	**3911.0 (1805.0–7335.0)**	**4014.0 (1859.0–7219.0)**	**0.03**	**<500.0**
Fibrinogen [mg dL^−1^]	526.0 (431.0–781.0)	539.0 (439.0–817.0)	0.86	200.0–393.0
Platelets [×10^3^ µL^−1^]	269.0 (195.0–357.0)	276.5 (197.5–372.5)	0.43	130–400

^1^ Median value. ^2^ Interquartile range. **In bold: statistically significant differences**.

**Table 3:** Results of rotational thromboelastometry pre–post-red blood cell transfusion in the study group.

Parameter	Value, Me ^1^ (IQR ^2^) Pre-Transfusion	Value, Me (IQR) Post-Transfusion	*p*-Value	Reference Range
INTEM CT ^3^ [s]	192.0 (173.0–211.0)	193.0 (171.0–208.0)	0.37	100–240
INTEM CFT ^4^ [s]	43.0 (38.0–48.0)	45.0 (39.0–49.0)	0.05	30–110
INTEM AA ^5^ [°]	81.0 (80.0–83.0)	81.0 (80.0–83.0)	0.14	70–83
**INTEM MCF ^6^ [mm]**	**78.0 (73.0–81.0)**	**77.0 (73.0–81.0)**	**<0.01**	**50–72**
INTEM ML ^7^ [%]	3.0 (1.0–5.0)	3.0 (0.0–5.0)	0.71	0–15
INTEM LI30 ^8^ [%]	100.0 (100.0–100.0)	100.0 (100.0–100.0)	1.00	94–100
INTEM LI45 ^9^ [%]	98.0 (97.0–100.0)	99.0 (97.0–100.0)	0.23	-
EXTEM CT [s]	82.0 (69.0–92.0)	83.0 (70.0–95.0)	0.98	38–79
**EXTEM CFT [s]**	**45.0 (38.0–52.0)**	**45.0 (37.0–52.0)**	**<0.01**	**34–159**
EXTEM AA [°]	81.0 (80.0–82.0)	81.0 (79.0–82.0)	0.06	63–83
EXTEM MCF [mm]	73.0 (70.0–78.0)	72.0 (70.0–78.0)	0.63	50–72
**EXTEM ML [%]**	**30.0 (7.0–62.0)**	**19.5 (4.0–42.0)**	**<0.01**	**0–15**
**EXTEM LI30 [%]**	**88.0 (73.0–100.0)**	**96.5 (81.0–100.0)**	**<0.01**	**94–100**
**EXTEM LI45 [%]**	**72.5 (46.0–95.0)**	**82.5 (60.0–97.0)**	**<0.01**	**-**
FIBTEM CT [s]	76.0 (67.0–90.0)	78.0 (63.0–91.0)	0.43	38–62
**FIBTEM CFT [s]**	**55.5 (45.0–89.0)**	**59.5 (48.0–118.0)**	**<0.01**	**≤300**
FIBTEM AA [°]	81.0 (78.0–82.0)	80.0 (77.0–81.0)	0.27	65–80
**FIBTEM MCF [mm]**	**35.0 (29.0–43.0)**	**33.0 (29.0–40.0)**	**<0.01**	**9–25**
FIBTEM ML [%]	0.0 (0.0–1.0)	0.0 (0.0–1.0)	0.52	0–15
FIBTEM LI30 [%]	100.0 (100.0–100.0)	100.0 (100.0–100.0)	1.00	-
FIBTEM LI45 [%]	100.0 (100.0–100.0)	100.0 (100.0–100.0)	0.37	-
APTEM CT [s]	80.0 (68.0–98.0)	79.0 (66.0–93.0)	0.75	33–62
APTEM CFT [s]	43.0 (37.0–49.0)	46.0 (38.0–54.0)	0.07	48–127
APTEM AA [°]	82.0 (80.0–82.0)	82.0 (79.0–82.0)	0.24	-
**APTEM MCF [mm]**	**78.0 (74.0–80.0)**	**76.0 (72.0–80.0)**	**0.04**	**61–79**
APTEM ML [%]	6.0 (3.0–9.0)	5.0 (2.0–10.0)	0.31	0–15
APTEM LI30 [%]	99.0 (98.0–100.0)	100.0 (98.0–100.0)	0.56	-
APTEM LI45 [%]	97.0 (94.0–98.0)	97.0 (93.0–99.0)	0.33	-
**MCFEXTEM-FIBTEM [mm]**	**37.0 (33.0–42.0)**	**40.0 (35.0–44.0)**	**<0.01**	**26–63**

^1^ Median value. ^2^ Interquartile range. ^3^ Clotting time. ^4^ Clot formation time. ^5^ Alpha angle. ^6^ Maximal clot firmness. ^7^ Maximal lysis. ^8^ Lysis index at 30 min. ^9^ Lysis index at 45 min. **In bold: statistically significant differences**.

**Table 4:** Changes in rotational thromboelastometry parameters pre–post-red blood cell transfusion in the subgroups of patients that received fresher (up to 14 days) and older (over 14 days) red blood cells.

Parameter	Change in Pre–Post-Transfusion Value, Me ^1^ (IQR ^2^) Fresher RBCs ^3^	Change in Pre–Post-Transfusion Value, Me (IQR) Older RBCs	*p*-Value
INTEM CT ^4^ [s]	9.5 (−7.5–35.5)	−4.0 (−17.0–4.0)	0.06
INTEM CFT ^5^ [s]	1.0 (−0.5–2.5)	1.0 (−1.0–3.0)	0.93
INTEM AA ^6^ [°]	0.0 (−0.5–0.0)	0.0 (−1.0–0.0)	0.91
INTEM MCF ^7^ [mm]	−1.0 (−2–(−0.5)	−1.0 (−2.0–0.0)	0.56
INTEM ML ^8^ [%]	0.0 (−0.5–0.0)	0.0 (−1.0–1.0)	0.82
INTEM LI30 ^9^ [%]	0.0 (0.0–0.0)	0.0 (0.0–0.0)	1.00
INTEM LI45 ^10^ [%]	0.0 (0.0–1.0)	0.0 (0.0–0.5)	0.68
EXTEM CT [s]	1.0 (−1.0–4.5)	−1.0 (−4.0–4.0)	0.40
EXTEM CFT [s]	3.5 (1.0–4.0)	1.0 (−1.0–5.0)	0.54
EXTEM AA [°]	0.0 (0.0–0.0)	0.0 (−1.0–0.0)	0.45
EXTEM MCF [mm]	0.0 (−2.0–1.5)	0.0 (−1.0–1.0)	0.88
EXTEM ML [%]	−13.5 (−24.0–1.5)	−7.0 (−15.0–0.0)	0.70
EXTEM LI30 [%]	1.0 (−1.5–8.5)	2.5 (0.0–13.0)	0.46
EXTEM LI45 [%]	17.0 (−2.0–24.0)	7.0 (0.0–15.0)	0.43
**FIBTEM CT [s]**	**2.0 (0.5–4.5)**	**−3.0 (−6.0–1.0)**	**0.03**
FIBTEM CFT [s]	33.5 (7.0–39.5)	6.0 (2.0–12.0)	0.05
FIBTEM AA [°]	0.0 (−1.0–0.5)	0.0 (−2.0–1.0)	0.65
FIBTEM MCF [mm]	−1.0 (−2.0–(−0.5)	−2.0 (−3.0–0.0)	0.20
FIBTEM ML [%]	0.0 (0.0–0.0)	0.0 (0.0–0.0)	0.76
FIBTEM LI30 [%]	0.0 (0.0–0.0)	0.0 (0.0–0.0)	1.00
FIBTEM LI45 [%]	0.0 (0.0–0.0)	0.0 (0.0–0.0)	0.76
APTEM CT [s]	1.5 (−11.0–5.5)	0.0 (−6.0–2.0)	0.62
APTEM CFT [s]	0.5 (−1.0–3.5)	3.0 (−1.0–5.0)	0.63
APTEM AA [°]	−0.5 (−1.0–0.5)	0.0 (−1.0–0.0)	0.84
APTEM MCF [mm]	−0.5 (−2.5–1.0)	−1.0 (−2.0–1.0)	0.92
APTEM ML [%]	−1.5 (−3.5–(−0.5)	0.0 (−2.0–3.0)	0.23
APTEM LI30 [%]	0.5 (−0.5–1.0)	0.0 (−1.0–1.0)	0.67
APTEM LI45 [%]	2.0 (0.0–2.0)	0.0 (−1.5–1.5)	0.31
MCFEXTEM-FIBTEM [mm]	2.0 (−1.0–2.5)	2.0 (0.0–4.0)	0.67

^1^ Median value. ^2^ Interquartile range. ^3^ Red blood cells. ^4^ Clotting time. ^5^ Clot formation time. ^6^ Alpha angle. ^7^ Maximal clot firmness. ^8^ Maximal lysis. ^9^ Lysis index at 30 min. ^10^ Lysis index at 45 min. **In bold: statistically significant differences**.

**Table 5:** Multivariable linear regression analysis of predictors of change in FIBTEM MCF post-red blood cell transfusion.

FIBTEM MCF ^1^ Change	Coefficient (95% CI ^2^)	*p*-Value
Red blood cell age	−0.09 (−0.26–0.08)	0.28
Blood volume change	−0.03 (−1.47–1.41)	0.96
Admission diagnosis	−0.09 (−1.26–1.08)	0.88

^1^ Maximal clot firmness. ^2^ Confidence interval.

**Table 6:** Multivariable linear regression analysis of predictors of change in MCF EXTEM-FIBTEM post-red blood cell transfusion.

MCF ^1^ EXTEM-FIBTEM Change	Coefficient (95% CI ^2^)	*p*-Value
Red blood cell age	−0.00 (−0.12–0.11)	0.95
Blood volume change	−0.51 (−2.37–1.35)	0.57
Admission diagnosis	−0.23 (−1.24–0.79)	0.64
Platelet number change	0.00 (−0.04–0.04)	0.97
**FIBTEM MCF change**	**−0.86 (−1.14–(−0.58)**	**<0.01**

^1^ Maximal clot firmness. ^2^ Confidence interval. **In bold: statistically significant differences**.

## Data Availability

The raw data supporting the conclusions of this article will be made available by the authors on request.
